# Accelerated long-term forgetting as an objective marker of subjective memory impairment in multiple sclerosis

**DOI:** 10.1186/s42466-026-00527-y

**Published:** 2026-08-24

**Authors:** Christina Jansen, Johannes Stalter, Sigrid Reuter, Karsten Witt

**Affiliations:** 1 Department of Neurology, Faculty of Medicine and Health Science , Carl von Ossietzky University of Oldenburg, Heiligengeisthöfe 4, Oldenburg, 26121 Germany; 2 Department of Neurology, Evangelical Hospital, Steinweg 13-17, Oldenburg, 26122 Germany; 3 Research Center of Neurosensory Science, Carl von Ossietzky University of Oldenburg, Carl-von-Ossietzky-Straße 9-11, Oldenburg, 26129 Germany

**Keywords:** Accelerated long-term forgetting, Multiple sclerosis, Subjective memory impairment, Cognitive dysfunction, Cognitive assessment

## Abstract

**Background:**

Accelerated long-term forgetting (ALF), defined as an increased rate of memory loss over extended intervals, has so far been detected in a pilot study of patients with mild multiple sclerosis (MS). This study aimed to (I) confirm the presence of ALF in a larger, heterogeneous MS sample, (II) explore associations with patient-reported outcomes, and (III) assess the diagnostic performance of ALF tests for subjective memory impairment.

**Methods:**

This study compared 62 MS patients and 65 age-, sex-, and education-matched healthy controls using standardized memory tests (RAVLT, WMS-IV Logical Memory subtest). Recall was assessed immediately, after 30 min, and after 7 days. Seven-day/30-minute recall ratios (Q_RAVLT_, Q_WMS_) served as primary outcomes. Self-report measures included memory complaints, fatigue, depression, and sleep disturbances. Spearman correlations and Receiver operating characteristic (ROC) analyses assessed associations and diagnostic accuracy.

**Results:**

ALF was observed in multiple sclerosis since Q_RAVLT_ was lower in patients than in controls (0.64 [95% CI 0.59–0.69] vs. 0.78 [0.73–0.82], *p* < 0.001), as was Q_WMS_ (0.79 [95% CI 0.74–0.84] vs. 0.95 [0.90–1.00], *p* < 0.001), despite comparable initial learning. Greater fatigue, higher memory complaints, sleep disturbances, older age, and greater disability were associated with lower ALF scores. The combined ALF score moderately discriminated subjective memory impairment (AUC 0.74; sensitivity 0.73; specificity 0.73).

**Conclusion:**

MS patients showed ALF despite normal initial learning, indicating a specific memory deficit undetected by standard tests. Long-delay recall using RAVLT and WMS-IV Logical Memory subtest may improve cognitive impairment detection in MS.

**Trial registration:**

German Clinical Trials Register (DRKS00035204), registered 17 April 2025.

**Supplementary Information:**

The online version contains supplementary material available at 10.1186/s42466-026-00527-y.

## Introduction

Cognitive impairments, particularly memory deficits, are common in multiple sclerosis (MS), a chronic autoimmune-mediated neurological disease [1]. These deficits can substantially affect patients’ quality of life, yet in some cases they can be difficult to detect in routine clinical assessments [2]. A specific type of memory impairment, accelerated long-term forgetting (ALF), is characterized by an increased rate of memory loss over extended intervals despite intact initial learning and recall [3]. The optimal delay interval for assessing ALF remains uncertain. Previous studies in patients with epilepsy have used retention intervals ranging from less than 24 h to several weeks after the initial learning session [4]. As standard memory tests typically assess recall only after short delays, ALF may remain undetected in clinical practice. This phenomenon has been well-documented in temporal lobe epilepsy [5]. More recently, it has also been identified in other neurological conditions such as traumatic brain injury (TBI), transient ischemic attack (TIA) and minor stroke as well as limbic encephalitis [6]. Weston et al. [7] were also able to detect ALF in presymptomatic carriers of Alzheimer’s disease mutations by analyzing the ratio of 7-day to 30-minute delayed recall performance on list, story, and figure memory tests. The same method was applied by Stalter et al. [8] in a small cohort of mildly impaired MS patients, where ALF was also demonstrated, particularly affecting verbal memory. However, the presence and clinical relevance of ALF in larger, more heterogeneous MS populations remain unclear.

Additionally, MS patients frequently report subjective memory impairment, which often lacks objective verification [9]. Dementia research has shown that cognitive decline occurs on a continuum, ranging from subclinical changes, to subjective cognitive deficits, then to mild cognitive impairment, and ultimately to clinical evident dementia [10]. Stalter et al. [8] suggested that ALF may provide an objective correlate for self-reported memory difficulties and fatigue.

This study aims to (I) determine the presence of ALF in a larger, clinically diverse cohort of MS patients, (II) explore the relationship between ALF and patient-reported outcomes, including SMI, fatigue, depression, and sleep disturbances, and (III) evaluate the diagnostic performance of the newly established ALF assessment method, using the RAVLT and the WMS-IV Logical Memory subtest, to discriminate subjective memory impairment.

## Materials and methods

### Selection and description of participants

Patients with MS were recruited at the Evangelical Hospital and its university outpatient clinic in Oldenburg, Germany. Eligible participants were ≥ 18 years, native German speakers, and diagnosed with MS (RRMS, SPMS, or PPMS) according to the 2017 revised McDonald criteria [11]. Exclusion criteria comprised the use of any CNS-active medication, including corticosteroids, within six weeks prior to enrollment, as well as any neurological or psychiatric comorbidity besides MS. In addition, a Montreal Cognitive Assessment (MoCA) score < 26 and a Beck Depression Inventory II (BDI - II) score ≥ 20 were exclusion criteria [12, 13]. Participants who did not complete the assessment were also excluded from the analyses. Healthy controls were matched for age, sex, and years of education and were, along with the given criteria, required to have no neurological and psychiatric conditions. Demographical information was obtained via self-report. Written informed consent was obtained from all participants prior to enrollment. The study was approved by the Ethics Committee of the Carl von Ossietzky University in Oldenburg, Germany (approval number 2024 − 163) and is registered in the German Clinical Trials Register (DRKS00035204).

### Data collection and measurements

Verbal memory was assessed using Rey’s Auditory Verbal Learning Test (RAVLT) and the Logical Memory subtest of the Wechsler Memory Scale-IV (WMS-IV).

The RAVLT contains a list of 15 words that the participant learns within 5 trials. After an interference list, the memorized terms are reproduced (0–15 points) [14]. The WMS-IV Logical Memory subtest consists of two stories from which as many details as possible should be remembered (0–50 points) [15]. A delayed recall follows after 30 min.

To assess ALF, an additional 7-day-delayed recall was obtained via telephone interview. The 7-day delayed recall assessment was conducted using standardized instructions and the same assessment procedures for all participants. Baseline and delayed recall assessments were conducted by the same examiner, who was not blinded to participant status. All participants were instructed to complete the assessment in a calm setting without distractions. Primary outcomes were the 7-day/30-minute delayed recall ratios for the RAVLT (*Q*_RAVLT_) and WMS-IV Logical Memory (*Q*_WMS_), following the methodology used in earlier studies [7, 8]. Only complete cases were included in the analyses.

Additional neurocognitive and neuropsychiatric measures included the MoCA, Symbol Digit Modalities Test (SDMT), and questionnaires including the Fatigue Impact Scale (FIS), BDI - II, Pittsburgh Sleep Quality Index (PSQI) and Epworth Sleepiness Scale (ESS) [12, 13, 16–19]. Subjective memory difficulties were evaluated with the Everyday Memory Questionnaire (EMQ) and two questionnaires focusing on memory and attention deficits in everyday life (FEAG, FEDA) [20–22]. In accordance with previous work, self-reported memory assessment was completed using two different methods. The participants were asked to report whether they currently experienced increased forgetfulness (yes/no) and to rate their perceived forgetfulness on a scale from 0 (not forgetful) to 100 (very forgetful) [8].

### Statistical methods

Differences in MS patients and healthy controls were analyzed using Chi-square or Fisher’s exact test in categorical demographic variables. Continuous demographic variables, neuropsychological, and questionnaire data were compared using the Mann-Whitney-U-test. T2 lesion load was assessed on MRI and categorized as low (< 10 lesions), moderate (10–19 lesions), or high (≥ 20 lesions) based on the number of T2-weighted lesions of the last MRI of the brain available. For the two primary outcomes, *Q*_RAVLT_ and *Q*_WMS_, Bonferroni correction was applied (α = 0.025). To address potential instability of ratio-based retention measures, a sensitivity analysis using analysis of covariance (ANCOVA) was performed. Seven-day recall performance was included as the dependent variable, group as a predictor, and 30-minute recall performance as a covariate. Spearman correlation coefficients were calculated to explore associations between Q_RAVLT_ and Q_WMS_ scores and self-reported measures (Subjective Memory Impairment, BDI-II, FEDA, FEAG, EMQ, ESS, PSQI, FIS) as well as MoCA, SDMT, Expanded Disability Status Scale (EDSS), disease duration (time since manifestation and time since diagnosis), and age. Correlation analyses were considered exploratory. P-values were adjusted for multiple comparisons using the Benjamini-Hochberg false discovery rate (FDR). The combined ALF score was calculated as the mean of the Q_RAVLT_ and the Q_WMS_ score. Receiver operating characteristic (ROC) analysis of the combined ALF score was conducted to evaluate diagnostic accuracy in discriminating subjective memory impairment, assessed by asking patients whether they had recently perceived themselves as more forgetful, reporting area under the curve (AUC), 95% confidence interval, optimal cut-off (based on the Youden Index), sensitivity, and specificity. All analyses and graphical visualizations were performed in RStudio (Version 2024.04.2 + 764) [23].

## Results

### Sample characteristics

**Fig. 1:**
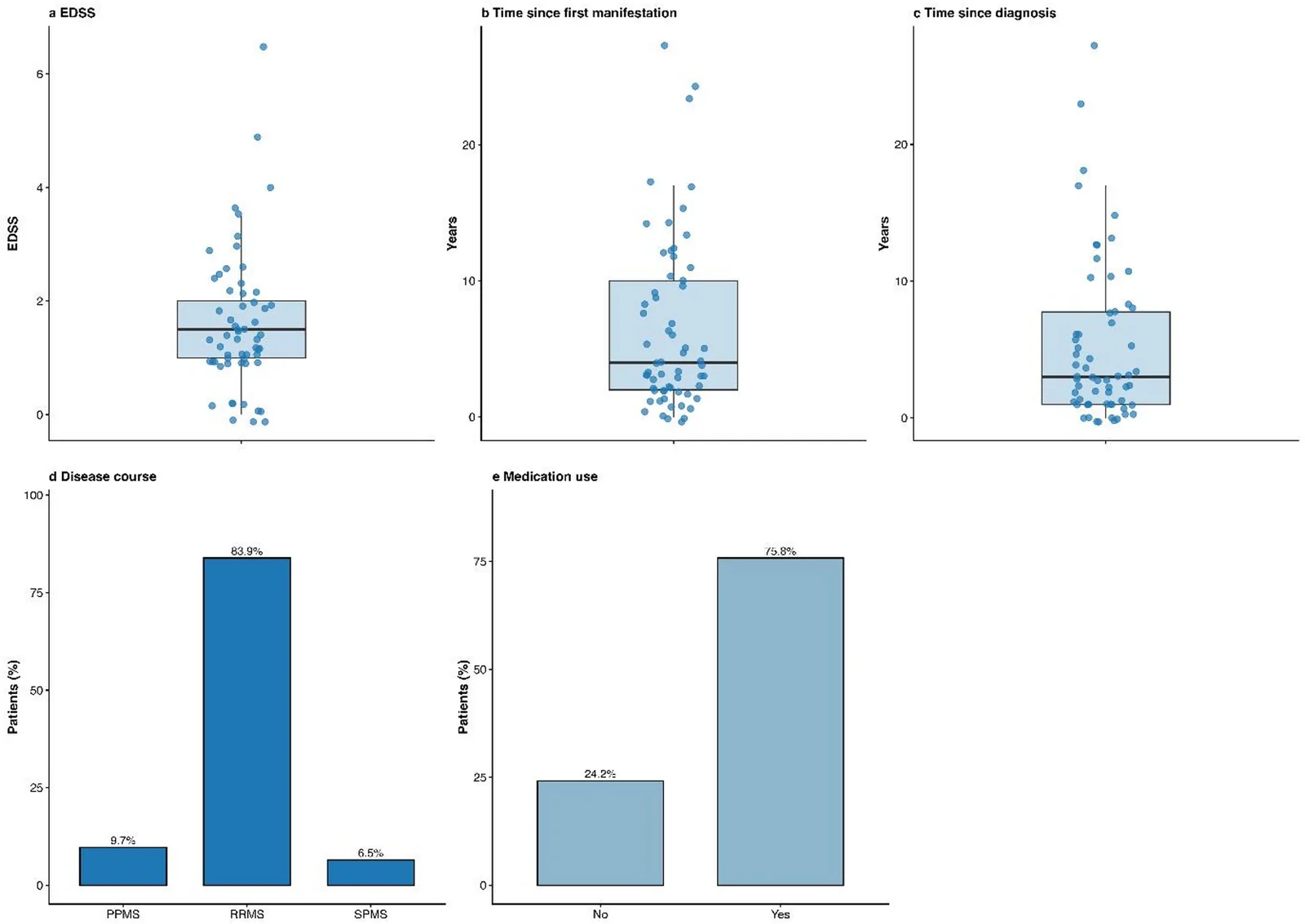
MS - specific characteristics. Distribution of Expanded Disability Status Scale scores (**a**), time since first manifestation (**b**), time since diagnosis (**c**), disease course (**d**), and disease-modifying medication use (**e**). Boxplots show the median and interquartile range with individual data points overlaid. Bar plots indicate the percentage of patients in each category. EDSS Expanded Disability Status Scale; PPMS Primary Progressive Multiple Sclerosis; RRMS Relapsing Remitting Multiple Sclerosis; SPMS Secondary Progressive Multiple Sclerosis

Seventy-six MS patients and 67 healthy controls were initially enrolled in the study. After exclusion of participants, due to failure to complete the delayed recall assessment (*n* = 3), MoCA-Score < 26 (*n* = 8), BDI-II-score ≥ 20 (*n* = 4), or treatment with CNS-active medication (*n* = 2), 62 MS patients and 65 healthy controls (HC) were included for the analyses. Demographic and clinical characteristics of the study population are summarized in Table 1. Figure 1 presents MS-specific characteristics. Additional information on disease-modifying medication used by MS patients is provided in Table A.1. Demographic characteristics did not differ significantly between both groups. Age, sex distribution, and MS phenotypes in the patient sample were roughly consistent with epidemiologic characteristics reported for MS populations [24].

**Table 1:** Demographical and clinical characteristics

	MS group (n = 62)	HC group (n = 65)	p-value
Sex (m/f)	12/50	15/50	0.768 (Chi^2^-test)
Mean age (min-max; SD)	37.03 (20–70; 13.38)	37.31 (18–66; 13.27)	0.856 (MWU)
Mean length of education (SD)	11.94 (1.33)	11.77 (1.36)	0.394 (MWU)
School leaving certificate			0.855 (FET)
Middle School	2	3	
Junior Highschool	18	21	
A-levels	42	41	
T2 lesion load (available n = 49)			
< 10 lesions (low)	19		
10 - 19 lesions (moderate)	12		
≥ 20 lesions (high)	18		
Mean time since last MRI, months (available n = 49; SD)	19.51 (19.06)		
Mean time since last relapse, months (available n = 56; SD)	35.36 (46.99)		

MS multiple sclerosis; HC healthy controls; n number of patients; m male; f female; min minimum; max maximum; SD standard deviation; MWU Mann-Whitney-U-test; FET Fisher’s exact test

### Primary outcomes

**Fig. 2:**
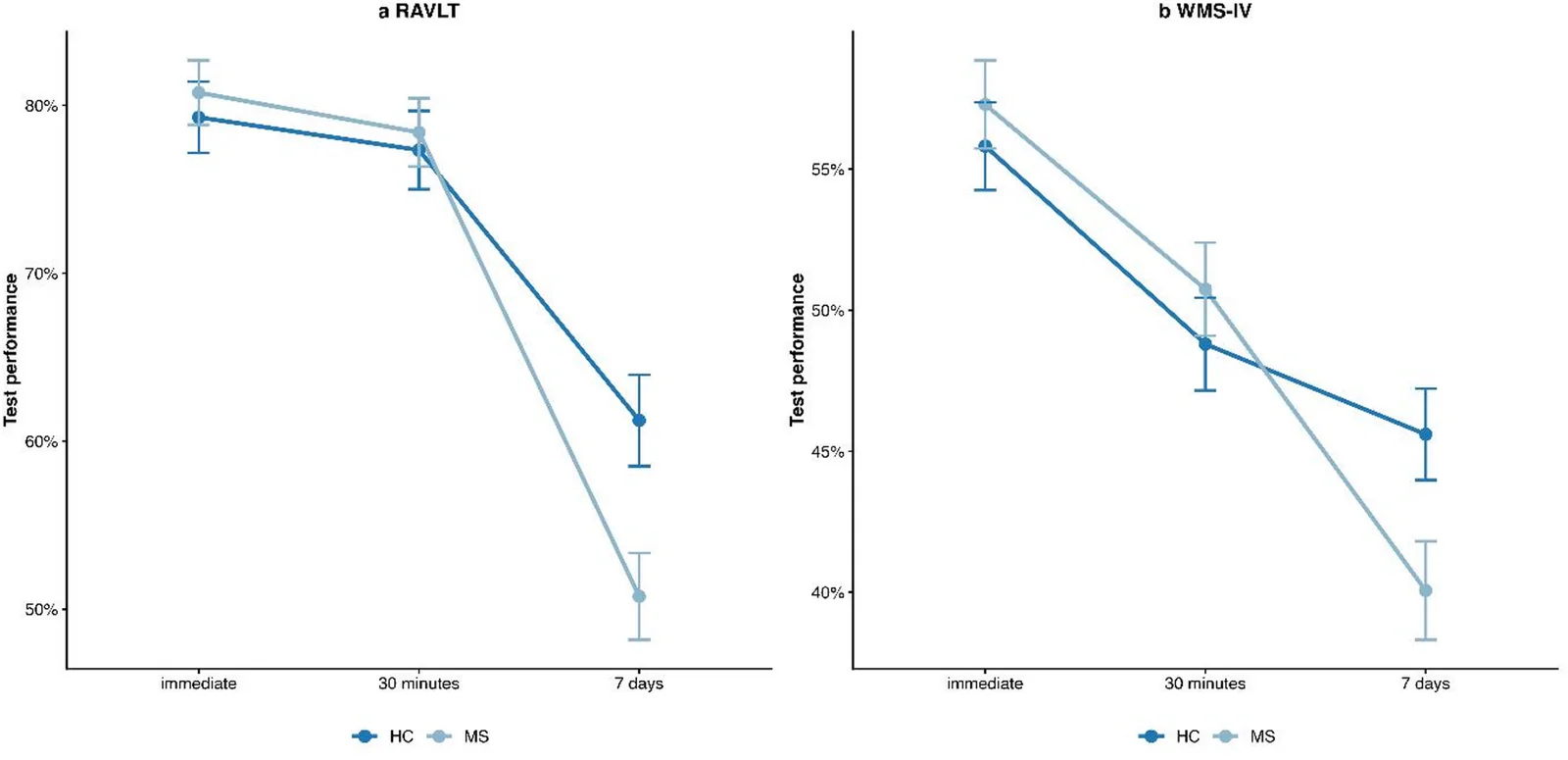
Performance trajectories across delay intervals. Mean (± SE) test performance (%) for the RAVLT (**a**) and WMS-IV Logical Memory subtest (**b**) in each group at the various test times. SE standard error; RAVLT Rey’s Auditory Verbal Learning Test; WMS Wechsler Memory Scale; HC healthy controls; MS multiple sclerosis

**Fig. 3:**
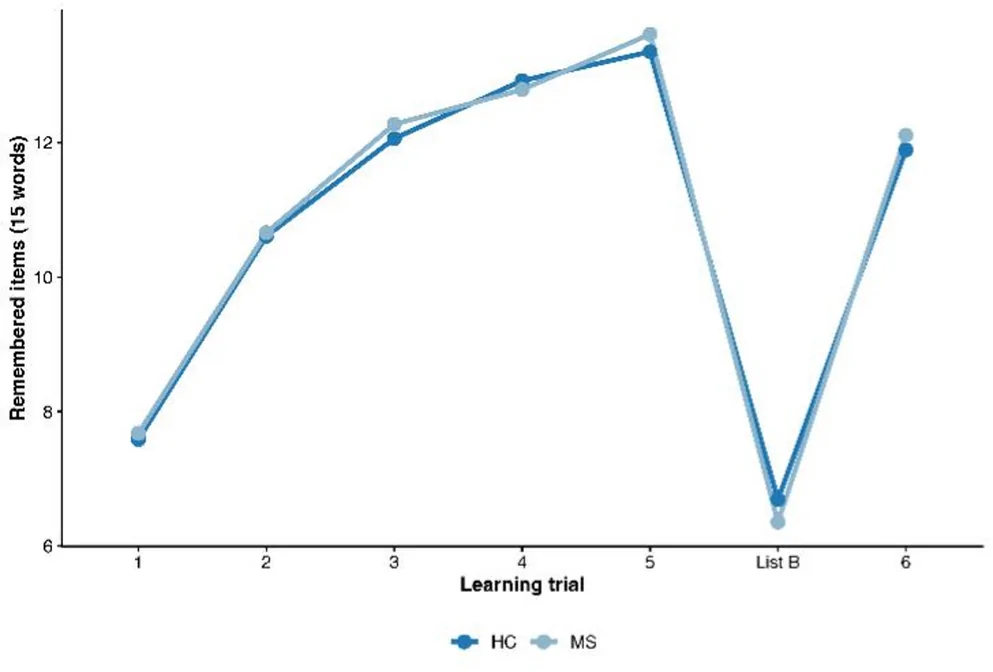
Trial-by-trial learning curves of the RAVLT. Learning curves of the mean number of remembered items in each group for the RAVLT. RAVLT Rey’s Auditory Verbal Learning Test; HC healthy controls; MS multiple sclerosis

Across both verbal memory tests, MS patients demonstrated significantly greater ALF than controls as measured with the described quotient. MS patients recalled fewer words after 7 days, resulting in a lower Q_RAVLT_ compared with HC (0.64 [95% CI 0.59–0.69] vs. 0.78 [0.73–0.82], *p* < 0.001). A similar pattern was observed for story recall: MS patients reproduced fewer story elements after 7 days, yielding lower Q_WMS_ scores (0.79 [95% CI 0.74–0.84] vs. 0.95 [0.90–1.0], *p* < 0.001). Immediate recall and 30-minute delayed recall did not differ between groups for either test. For both memory tests, the group effect remained significant after adjustment for 30-minute recall performance (RAVLT: B = −0.115, *p* < 0.001; WMS: B = −0.070, *p* < 0.001). The interaction between group and 30-minute recall performance was not significant for either test (RAVLT: *p* = 0.801; WMS: *p* = 0.690), indicating that the group differences were not dependent on the 30-minute recall performance. Complete results for the RAVLT and WMS-IV Logical Memory subtest are presented in Table 2 with performance trajectories across delay intervals shown in Fig. 2. Initial Trial-by-trial learning curves for the RAVLT did not differ between groups, as shown in Fig. 3. Mean scores across RAVLT trials are provided in Table A.2.

**Table 2:** ALF test results

	MS group (*n* = 62)	HC group (*n* = 65)	*p*-value
Results after initial testing			
RAVLT initial score (%, min-max)	80.75 (40–100)	79.28 (26.67–100)	0.924 (MWU)
WMS-IV initial score (%, min-max)	57.29 (28–84)	55.82 (28–84)	0.457 (MWU)
Results after 30 min			
RAVLT 30-minute-score (%, min-max)	78.39 (46.67–100)	77.33 (20–100)	0.923 (MWU)
WMS-IV 30-minute-score (%, min-max)	50.74 (18–80)	48.80 (18–78)	0.508 (MWU)
Results after 7 days			
RAVLT 7-day-score (%, min-max)	50.75 (6.67–93.33)	61.23 (13.33–100)	0.004 (MWU)
WMS-IV 7-day-score (%, min-max)	40.06 (10–78)	45.60 (12–70)	0.007 (MWU)
Q_RAVLT_ (min-max)	0.64 (0.13–1.13)	0.78 (0.29–1.22)	< 0.001 (MWU)
Q_WMS_ (min-max)	0.79 (0.19–1.12)	0.95 (0.32–1.50)	< 0.001 (MWU)

MS multiple sclerosis; HC healthy controls; n number of patients; RAVLT Rey’s Auditory Verbal Learning Test; WMS Wechsler Memory Scale; min minimum; max maximum; MWU Mann-Whitney-U-test

### Additional neurocognitive test results and self-reported symptoms

**Fig. 4:**
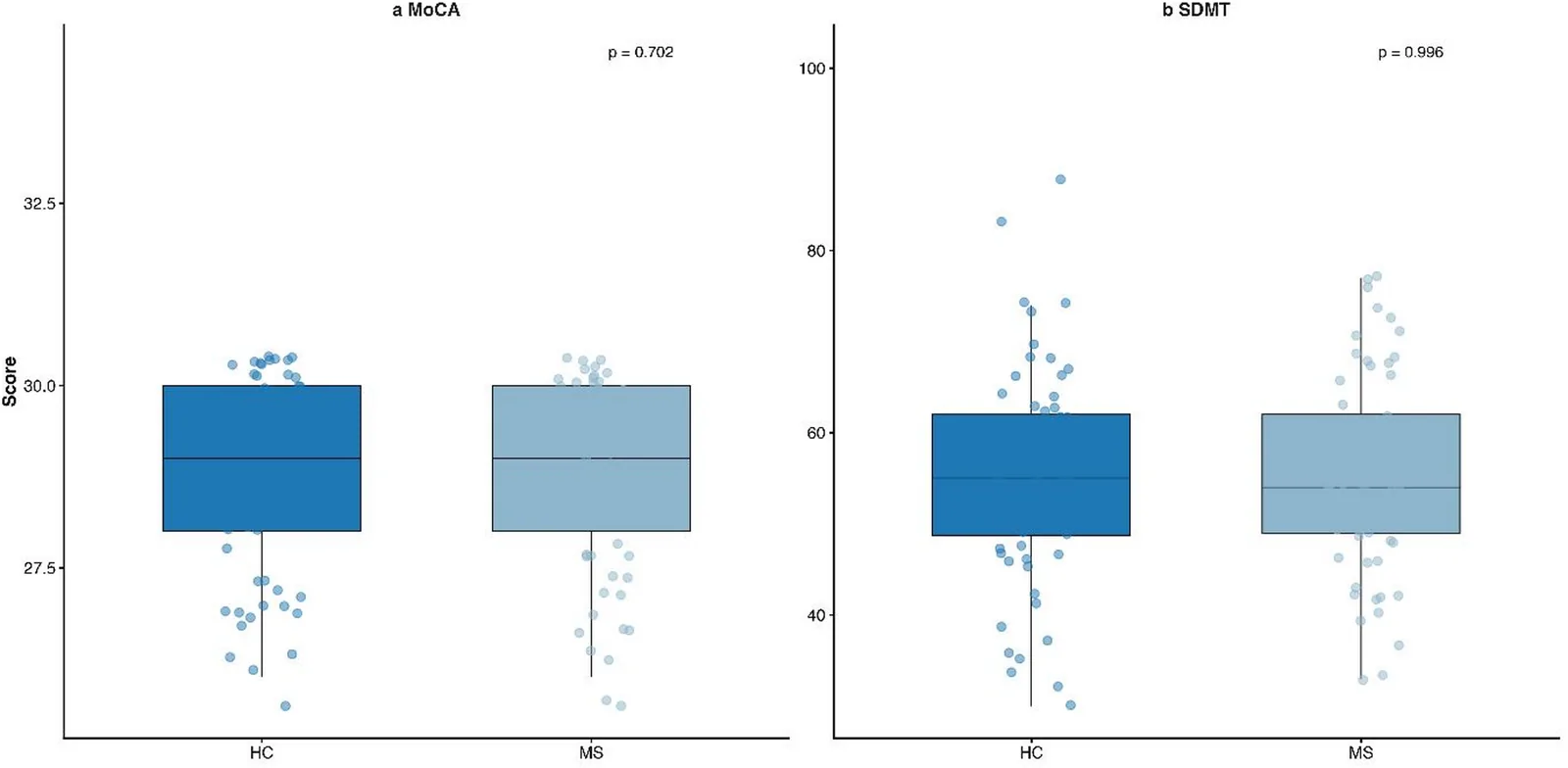
MoCA and SDMT results. Comparison of MoCA (**a**) and SDMT (**b**) scores between patients and healthy controls. Boxplots show the median and interquartile range with individual data points overlaid. HC healthy controls; MS multiple sclerosis; MoCA Montreal Cognitive Assessment; SDMT Symbol Digit Modalities Test

**Fig. 5:**
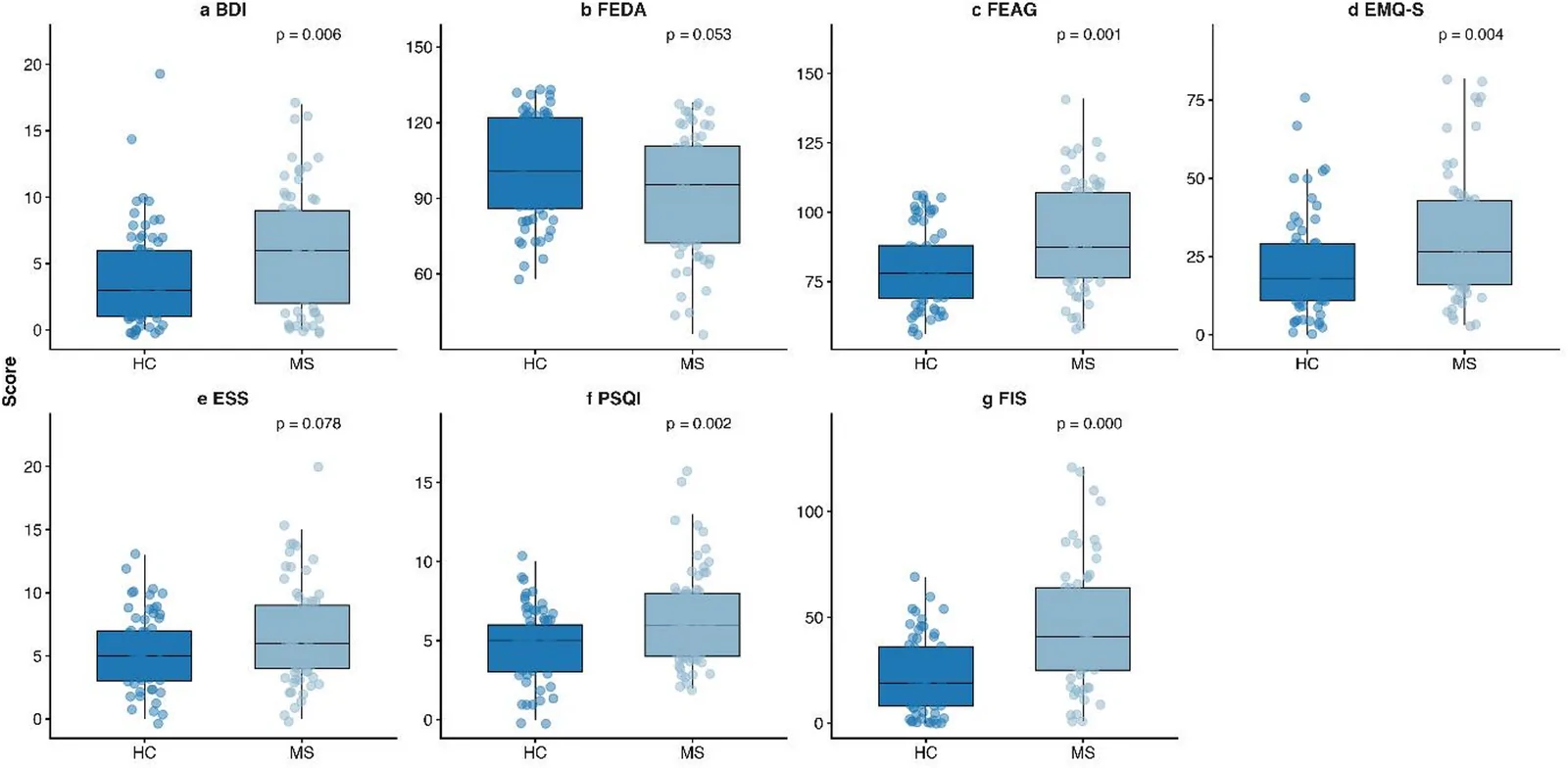
Questionnaire scores. Comparison of questionnaire scores between patients and healthy controls. Boxplots show the median and interquartile range with individual data points overlaid. HC healthy controls; MS multiple sclerosis; BDI Beck-Depression-Inventory II (**a**); FEDA Questionnaire of Experienced Deficits of Attention (**b**); FEAG Everyday Memory Assessment Questionnaire (**c**); EMQ-S Everyday Memory Questionnaire (**d**); ESS Epworth Sleepiness Scale (**e**); PSQI Pittsburgh Sleep Quality Index (**f**); FIS-D Fatigue Impact Scale (**g**)

Global cognition (MoCA) and information processing speed (SDMT) did not show significant differences between both groups as shown in Fig. 4. MS patients self-reported significantly greater fatigue (FIS) and poorer sleep quality (PSQI) than control participants. Subjective memory complaints, as assessed using the EMQ and FEAG, were significantly higher in MS patients. All questionnaire scores are shown in Fig. 5.

When asked whether they currently experienced increased forgetfulness (yes/no), MS patients reported this significantly more often than controls (22/62 vs. 7/65, *p* = 0.002). On a scale from 0 (not forgetful) to 100 (very forgetful), MS patients also reported greater forgetfulness (37.26 ± 16.51 vs. 29.23 ± 15.34, *p* = 0.003).

All results of MoCA, SDMT, and self-reported measures are summarized in Table A.3.

### Spearman correlation analyses

Within the MS group, fatigue severity was significantly associated with Q_RAVLT_ performance. For Q_WMS_ performance, significant associations were observed with fatigue severity, sleep disturbances, subjective cognitive complaints (SMI [yes/no], FEDA, FEAG, EMQ), disability (EDSS), and age. No significant associations were identified within the HC group. Detailed information on the correlation analyses is provided in Tables 3 and 4.

**Table 3:** Spearman correlation coefficients in the MS group

	Q_RAVLT_			Q_WMS_		
	ρ	*p*-value	adjusted *p*-value	ρ	*p*-value	adjustedp-value
SMI (0–100)	−0.215	0.093	0.174	−0.224	0.080	0.100
SMI (yes/no)	−0.334	0.008	0.053	−0.396	0.001	0.004
MoCA	0.189	0.141	0.209	0.139	0.282	0.282
SDMT	0.132	0.312	0.334	0.277	0.030	0.050
BDI	−0.133	0.303	0.334	−0.215	0.093	0.107
FEDA	0.309	0.014	0.053	0.516	< 0.001	0.004
FEAG	−0.290	0.022	0.060	−0.430	< 0.001	0.004
EMQ-S	−0.318	0.012	0.053	−0.390	0.002	0.005
ESS	−0.184	0.153	0.209	−0.167	0.195	0.209
PSQI	−0.208	0.105	0.175	−0.345	0.006	0.129
FIS	−0.425	< 0.001	0.015	−0.452	< 0.001	0.038
EDSS	−0.247	0.060	0.129	−0.344	0.008	0.015
Time since manifestation	−0.094	0.466	0.466	−0.246	0.054	0.074
Time since diagnosis	−0.138	0.285	0.334	−0.265	0.038	0.057
Age	−0.287	0.024	0.060	−0.386	0.002	0.005

RAVLT = Rey’s Auditory Verbal Learning Test; WMS = Wechsler Memory Scale IV; ρ = Spearman correlation coefficient; SMI Subjective Memory Impairment; MoCA Montreal Cognitive Assessment; SDMT Symbol Digit Modalities Test; BDI Beck-Depression-Inventory II; FEDA Questionnaire of Experienced Deficits of Attention; FEAG Everyday Memory Assessment Questionnaire; EMQ-S Everyday Memory Questionnaire; ESS Epworth Sleepiness Scale; PSQI Pittsburgh Sleep Quality Index; FIS-D Fatigue Impact Scale; EDSS Expanded Disability Status Scale

**Table 4:** Spearman correlation coefficients in the control group

	Q_RAVLT_		Q_WMS_	
	ρ	*p*-value	ρ	*p*-value
SMI (0–100)	−0.136	0.280	−0.130	0.301
SMI (yes/no)	−0.583	0.645	−0.069	0.586
MoCA	0.047	0.708	0.072	0.571
SDMT	0.050	0.697	0.191	0.131
BDI	−0.087	0.490	0.078	0.536
FEDA	0.169	0.177	0.185	0.140
FEAG	−0.164	0.190	−0.091	0.472
EMQ-S	−0.121	0.337	0.066	0.600
ESS	−0.176	0.160	−0.128	0.310
PSQI	0.100	0.426	0.139	0.268
FIS	−0.138	0.274	0.071	0.573
Age	0.018	0.885	−0.088	0.487

RAVLT = Rey’s Auditory Verbal Learning Test; WMS = Wechsler Memory Scale IV; ρ = Spearman correlation coefficient; SMI Subjective Memory Impairment; MoCA Montreal Cognitive Assessment; SDMT Symbol Digit Modalities Test; BDI Beck-Depression-Inventory II; FEDA Questionnaire of Experienced Deficits of Attention; FEAG Everyday Memory Assessment Questionnaire; EMQ-S Everyday Memory Questionnaire; ESS Epworth Sleepiness Scale; PSQI Pittsburgh Sleep Quality Index; FIS-D Fatigue Impact Scale

### Discriminatory accuracy

**Fig. 6:**
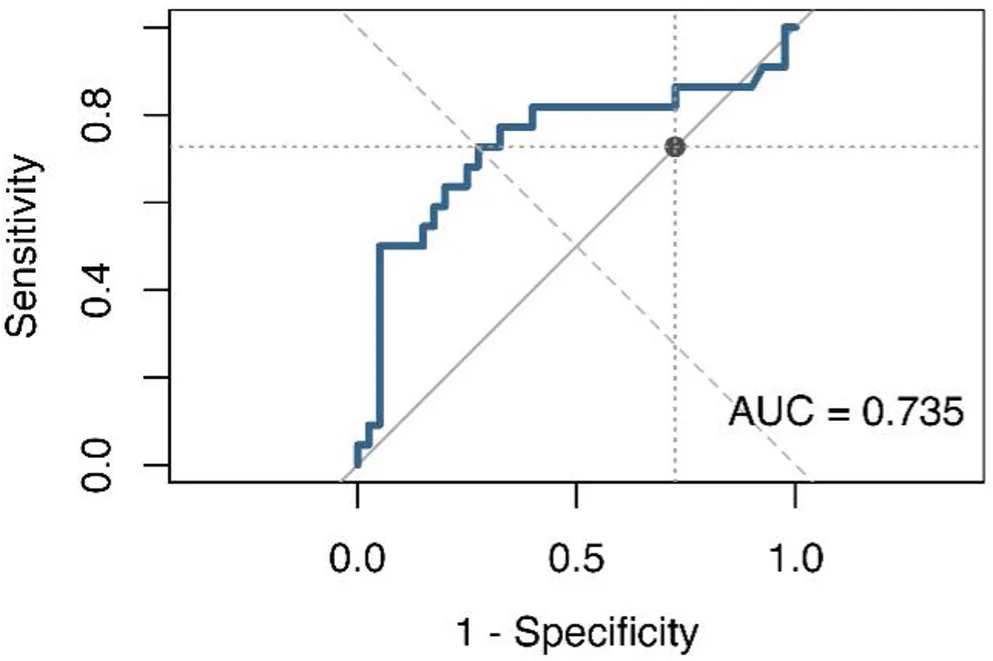
ROC curve of the combined ALF score for detecting subjective memory impairment

ROC analysis demonstrated moderate discriminatory accuracy of the combined ALF score for detecting subjective memory impairment (AUC = 0.735, 95% CI 0.585–0.884). The optimal cut-off value, determined using the Youden Index, was 0.71, yielding a sensitivity of 0.727 and a specificity of 0.725. The corresponding ROC curve is shown in Fig. 6.

Receiver operating characteristic (ROC) curve for the combined ALF score, illustrating the diagnostic accuracy in discriminating between presence vs. absence of subjective memory impairment. Sensitivity is plotted against the false-positive rate (1-specificity). The diagonal dotted line represents chance performance. The dot indicates the optimal cut-off based on the Youden Index. AUC area under the curve.

## Discussion

In this study, we administered the RAVLT and the WMS-IV Logical Memory subtest to a heterogeneous group of MS patients and matched healthy controls to replicate previously reported evidence of ALF in MS in a larger group of patients. In addition, correlation analyses were used to explore potential associations between ALF and other clinical measures within the patient group, and we examined the diagnostic performance of the RAVLT and the WMS-IV Logical Memory subtest for discriminating subjective memory impairment.

Consistent with the findings of Stalter et al. [8], we observed significantly lower ALF scores in MS patients when assessed with the word list (Q_RAVLT_). Beyond replicating these results, we were able to demonstrate significantly reduced ALF scores in the story-learning test (Q_WMS_). For both neuropsychological tasks, there was no difference between patients and controls in terms of immediate and short-delay recall or learning ability. These findings indicate that ALF in MS is caused by a problem in late consolidation rather than during initial encoding or early consolidation.

By applying these tests in a larger MS sample, we extend previous findings on ALF and provide additional support for our first hypothesis. Our results indicate that ALF affecting verbal memory is present in a patient population that resembles real-world clinical demographics, with the predominance of female patients and a relapsing-remitting disease course [24]. The materials used for this study met the requirements that Baddeley et al. [25] set for ALF assessment materials. Both tests provide reliable results when repeated, without requiring long learning periods. As the participants were not informed about the delayed recall assessment, strategic differences were minimized, and the tests also allowed for remote retesting [25]. Furthermore, we mostly followed the recommendations proposed by Elliott et al. [5] to ensure the quality of ALF assessment. Patients and controls were matched for age and intellectual ability, recall and recognition were tested, and distraction tasks ensured that the information was transferred to long-term memory. Ceiling and floor effects were reduced by matching initial learning, and practice of the test material prior to delayed retrieval was prevented [5].

With this study we extended the findings on ALF, suggesting that this specific cognitive impairment is also relevant in MS, aligning with findings from other neurological conditions [6]. ALF has previously been linked to disruptions in hippocampal-neocortical interaction and long-term consolidation mechanisms in patients with temporal lobe epilepsy (TLE) [3, 26]. While forgetting over short intervals is associated with hippocampal changes, its contribution to ALF remains poorly understood [26]. It is well established that the hippocampus is primarily involved in the early phase of memory formation, while neocortical structures become increasingly relevant over time [3]. Audrain and McAndrews [26] demonstrated that ALF in TLE patients is associated with reduced resting-state connectivity between the anterior hippocampus and the lateral temporal cortex. This finding supports the assumption that ALF cannot be completely explained by hippocampal changes, it rather reflects impaired interactions within hippocampal-neocortical networks.

Disconnection of the hippocampus from different brain networks, as well as focal hippocampal damage, is also known to be a contributing factor to cognitive impairment in MS [27]. These parallels suggest that ALF in MS may arise from similar disruptions in long-term consolidation pathways as those described in other neurological conditions. Inflammatory demyelination and neurodegenerative processes may interfere with the transfer of acquired information into neocortical storage sites. Future studies that extend the current methods by integrating structural and functional imaging could help clarify the neurobiological mechanisms underlying ALF in MS and determine whether ALF may be useful as an early cognitive marker for subtle network dysfunction.

Based on previous research, fatigue and self-perceived forgetfulness (rated on a 0–100 scale) have been proposed as potential correlates of ALF [8]. Spearman correlation analyses in our cohort indicated significant associations between ALF and these previously reported variables. Furthermore, additional associations were observed with sleep disturbances, EDSS, and age. Kinsinger et al. [28] demonstrated that fatigue influences subjective, but not objective measures of neuropsychological functioning. Combined with our findings, this may suggest that fatigue affects long-term memory formation and consolidation rather than short-term memory, which is typically assessed in standard neuropsychological testing. The comorbidity of fatigue and ALF could be explained because both are dependent on sleep quality, which is often impaired in MS patients [29]. Various types of sleep disturbances, including insomnia, restless legs syndrome and sleep related breathing disorders, have been associated with MS. The underlying cause may be primary insomnia, but MS-related symptoms, such as pain and spasticity, may also contribute to these conditions [30].

Prior evidence has shown that older age relates to cognitive impairment in multiple sclerosis, whereas the association with EDSS was only a trend [31]. Given that age is considered to be an important determinant of ALF, age-related effects may not be disease-specific [5]. Overall, these findings suggest that the cumulative effects of neurodegeneration may also contribute to impaired long-term memory consolidation.

Regarding subjective memory impairment, our results also suggest an association with ALF. In our study, this relationship was observed based on self-report questionnaires (FEAG, FEDA, EMQ) and participants’ responses to whether they perceived themselves as being more forgetful recently. However, these findings were observed only for the WMS-IV story recall test. Stalter et al. [8] reported comparable findings, although they found associations with memory complaints using a 0–100 self-rating scale. Our findings suggest that the measures used to assess perceived memory difficulties may need to be further improved to accurately predict objective memory impairment.

In summary, ALF testing may provide an objective complement to subjective memory complaints and fatigue-related difficulties. However, the questionnaires, rating scales, and binary questions employed in the present study appear insufficient for a comprehensive assessment of subjective memory impairment.

Measures of cognitive function have been identified as strong prognostic markers in MS, potentially providing information beyond conventional MRI measures. As cognitive decline may occur even during periods of apparent clinical remission, regular cognitive assessment could help identify inadequate disease control [32]. Regarding the diagnostic performance in discriminating subjective memory impairment, ROC analysis indicated moderate accuracy for the combined ALF score. Thus, the long-delay recall extensions of the RAVLT and the WMS-IV Logical Memory subtest appear suitable for identifying memory impairments that remain undetected in short-delay recall assessments. Given their sufficient sensitivity for detecting ALF, these methods allow the identification of individuals who may benefit from more extensive neuropsychological testing.

Acknowledging the fact that cognitive decline progresses continuously, with subjective memory complaints preceding mild cognitive impairment and eventually dementia, ALF measures may offer an approach for earlier detection and enable interventions that may positively influence symptom progression. Most interventions aim to strengthen cognitive reserve, thereby supporting brain function and potentially slowing cognitive decline [33]. This finding highlights the potential clinical value of detecting subtle memory deficits that standard neuropsychological tests may not recognize.

As we are using a cross-sectional design, we cannot provide information on the evolution of ALF over time. Additional longitudinal studies could help to determine whether ALF can also predict disease progression and future cognitive decline in MS patients.

Some limitations should be considered when interpreting our findings. We assessed ALF exclusively in the domain of verbal memory. Elliott et al. [5] recommend that the evaluation should include both verbal and nonverbal memory modalities. Although visuospatial memory ALF has been observed across different patient populations, it has not yet been demonstrated in patients with MS [3, 7, 34, 35]. One possible explanation is the negative association of the visuospatial memory with the EDSS as found by Artemiadis et al. [36], since the EDSS in our study was rather low. In their study, Stalter et al. [8] used Rey’s complex figure test to assess ALF in visuospatial memory. However, results did not differ between MS patients and controls, as both groups performed poorly on the 7-day delayed recall. Given these findings, future studies should consider using alternative methods to investigate ALF in visuospatial memory.

Several methodological limitations relate to the fact that the 7-day recall assessment was conducted by telephone. Standardized instructions and the same assessment procedure were used for all participants. Both baseline and delayed recall assessments were conducted by the same examiner. While this ensured consistency in test administration, the examiner was not blinded to participant status. Even though the participants were instructed to be in a calm setting without distractions, environmental conditions and potential distractions during the telephone assessment could not be fully standardized and may have influenced test performance.

A further limitation is that the generalizability of our results may be limited by the characteristics of our study cohort. The majority of patients had relapsing-remitting MS, while patients with progressive disease phenotypes were less represented. Although disease phenotype may influence cognitive performance, we were unable to investigate potential differences in ALF outcome measures between MS subtypes because the number of patients with progressive MS was too small to allow reliable subgroup analyses. Future studies including more diverse MS cohorts are needed to further evaluate the generalizability of our findings across different disease phenotypes.

Furthermore, the methods used to detect subjective memory impairment may limit the interpretation of our findings, as there is no standardized method for assessing self-experienced forgetfulness [37]. Abdulrab and Heun [37] recommend the specification of time-related details, providing examples, and obtaining confirmation through a relative. Future studies therefore should consider alternative measures for subjective cognitive deterioration, as current questionnaires and visual analogue scales may not be sufficient.

Other limitations involve the use of medication in the patient group. While we defined the use of medication with a proven effect on CNS-function as an exclusion criterion, 47 of the 62 patients used nonsteroid immune suppressive MS-medication. The effect of these drugs on memory function has not yet been sufficiently investigated. In addition, patients who had received recent corticosteroid treatment were excluded. Consequently, the generalizability of our findings is limited to patients with characteristics comparable to those in our study cohort.

MS is the most common cause of non-traumatic disability in young adults [38]. Cognitive deficits, such as objectively detectable and subjectively perceived memory impairments, contribute substantially to impairments in professional and private life [2]. Unlike many other chronic neurological diseases, MS usually manifests itself in young, active people, meaning that the symptoms of the disease accompany those affected for a long time [39]. Paving the way for reducing these relevant effects on patients is therefore the primary goal of this study. This work aims to contribute to improved neuropsychological evaluation by testing a methodology for the reliable ALF assessment, which has already been successful in previous studies [7, 8]. Establishing sensitive methods for the detection of ALF may provide access to therapeutic interventions for memory impairment at an early stage, thereby positively influencing the course of the disease.

## Supplementary Information

Supplementary Material 1

## Data Availability

Data supporting this study’s findings are available from the corresponding author upon reasonable request.

## References

[1] Benedict, R. H. B., Amato, M. P., DeLuca, J., & Geurts, J. J. G. (2020). Cognitive impairment in multiple sclerosis: Clinical management, MRI, and therapeutic avenues. *Lancet Neurology,**19*, 860–71. 10.1016/S1474-4422(20)30277-5 32949546 10.1016/S1474-4422(20)30277-5 PMC10011205

[2] Amato, M. P., Zipoli, V., & Portaccio, E. (2006). Multiple sclerosis-related cognitive changes: A review of cross-sectional and longitudinal studies. *Journal Of The Neurological Sciences*, *245*, 41–46. 10.1016/j.jns.2005.08.019 16643953 10.1016/j.jns.2005.08.019

[3] Mameniskiene, R., Puteikis, K., Jasionis, A., & Jatuzis, D. (2020). A Review of Accelerated Long-Term Forgetting in Epilepsy. *Brain Sci*, *10*. 10.3390/brainsci10120945 10.3390/brainsci10120945 PMC7762289 33297371

[4] Cassel, A., & Kopelman, M. D. (2019). Have we forgotten about forgetting? A critical review of ‘accelerated long-term forgetting’ in temporal lobe epilepsy. *Cortex; A Journal Devoted To The Study Of The Nervous System And Behavior*, *110*, 141–149. 10.1016/j.cortex.2017.12.012 29331203 10.1016/j.cortex.2017.12.012

[5] Elliott, G., Isaac, C. L., & Muhlert, N. (2014). Measuring forgetting: A critical review of accelerated long-term forgetting studies. *Cortex; A Journal Devoted To The Study Of The Nervous System And Behavior*, *54*, 16–32. 10.1016/j.cortex.2014.02.001 24631847 10.1016/j.cortex.2014.02.001 PMC4007031

[6] Butler, C., Gilboa, A., & Miller, L. (2019). Accelerated long-term forgetting. *Cortex; A Journal Devoted To The Study Of The Nervous System And Behavior*, *110*, 1–4. 10.1016/j.cortex.2018.12.009 30616746 10.1016/j.cortex.2018.12.009

[7] Weston, P. S. J., Nicholas, J. M., Henley, S. M. D., Liang, Y., Macpherson, K., Donnachie, E., et al. (2018). Accelerated long-term forgetting in presymptomatic autosomal dominant Alzheimer’s disease: A cross-sectional study. *Lancet Neurology*, *17*, 123–132. 10.1016/S1474-4422(17)30434-9 29413314 10.1016/S1474-4422(17)30434-9 PMC5958413

[8] Stalter, J., Pars, K., & Witt, K. (2024). Accelerated long-term forgetting reveals everyday memory deficits in early-stage multiple sclerosis. *Journal Of Neurology*. 10.1007/s00415-024-12359-4 38587635 10.1007/s00415-024-12359-4 PMC11233340

[9] Krch, D., Sumowski, J. F., DeLuca, J., & Chiaravalloti, N. (2011). Subjective memory in multiple sclerosis is associated with initial-trial learning performance. *Journal Of The International Neuropsychological Society*, *17*, 557–561. 10.1017/S1355617711000336 21411038 10.1017/S1355617711000336

[10] An, R., Gao, Y., Huang, X., Yang, Y., Yang, C., & Wan, Q. (2024). Predictors of progression from subjective cognitive decline to objective cognitive impairment: A systematic review and meta-analysis of longitudinal studies. *International Journal of Nursing Studies,**149*, Article 104629. 10.1016/j.ijnurstu.2023.104629 37979370 10.1016/j.ijnurstu.2023.104629

[11] Thompson, A. J., Banwell, B. L., Barkhof, F., Carroll, W. M., Coetzee, T., Comi, G., et al. (2018). Diagnosis of multiple sclerosis: 2017 revisions of the McDonald criteria. *Lancet Neurology,**17*, 162–73. 10.1016/S1474-4422(17)30470-2 29275977 10.1016/S1474-4422(17)30470-2

[12] Nasreddine, Z. S., Phillips, N. A., Bedirian, V., Charbonneau, S., Whitehead, V., Collin, I., et al. (2005). The Montreal Cognitive Assessment, MoCA: A brief screening tool for mild cognitive impairment. *Journal Of The American Geriatrics Society*, *53*, 695–699. 10.1111/j.1532-5415.2005.53221.x 15817019 10.1111/j.1532-5415.2005.53221.x

[13] Herzberg, P., Goldschmidt, S., & Heinrichs, N. (2008). Beck Depressions-Inventar (BDI-II). *Revision Rep Psychol*, *33*, 301–302.

[14] Bean, J. (2018). Rey Auditory Verbal Learning Test, Rey AVLT. In J. Kreutzer, J. DeLuca, & B. Caplan (Eds.), *Encyclopedia of Clinical Neuropsychology* (pp. 3020–2). Springer International Publishing.

[15] Lepach, A., & Petermann, F. (2012). Gedächtnisdiagnostik mit der Wechsler Memory Scale – Fourth Edition. *Zeitschrift Für Neuropsychologie,**23*, 123–32. 10.1024/1016-264X/a000070

[16] Smith, A. (1973). *Symbol Digit Modalities Test*. Western Psychological Services.

[17] Häuser, W., Almouhtasseb, R., Muthny, F., & Grandt, D. (2003). Validierung der deutschen version der fatigue impact scale FIS-D. *Zeitschrift für Gastroenterologie*, *41*, 973–982. 14562194 10.1055/s-2003-42927

[18] Buysse, D. J., Reynolds, C. F., 3rd., Monk, T. H., Berman, S. R., & Kupfer, D. J. (1989). The Pittsburgh sleep quality index: A new instrument for psychiatric practice and research. *Psychiatry Research,**28*, 193–213. 10.1016/0165-1781(89)90047-4 10.1016/0165-1781(89)90047-4 2748771

[19] Johns, M. W. (1991). A new method for measuring daytime sleepiness: The Epworth sleepiness scale. *Sleep*, *14*, 540–545. 1798888 10.1093/sleep/14.6.540

[20] Sunderland, A., Harris, J., & Baddeley, A. (1983). Assessing everyday memory after severe head injury. In J.E. Harris & P.E. Morris (Eds.), *Everyday Memory, Actions and Absentmindedness* (pp. 191–206). Academic Press.

[21] Hupfeld, J. (2009). Fragebogen zur Erfassung alltäglicher Gedächtnisleistungen (FEAG). In D. Schellig, R. Drechsler, D. Heinemann, & W. Sturm (Eds.), *Handbuch neuropsychologischer Testverfahren Aufmerksamkeit, Gedächtnis und exekutive Funktionen 1* (pp. 677–83). Hogrefe.

[22] Zimmermann, P., Messner, C., Poser, U., & Sedelmeier, P. (1991). Ein Fragebogen erlebter Defizite der Aufmerksamkeit (FEDA).

[23] Posit, R. S. (2024). *Integrated Development Environment for R. 2024.04.2 + 764 ed*. Posit Software, PBC.

[24] Multiple Sclerosis International Federation. (2020). *Atlas of MS 3rd Edition: Mapping multiple sclerosis around the world - key epidemiology findings*. Multiple Sclerosis International Federation.

[25] Baddeley, A., Atkinson, A., Kemp, S., & Allen, R. (2019). The problem of detecting long-term forgetting: Evidence from the Crimes Test and the Four Doors Test. *Cortex,**110*, 69–79. 10.1016/j.cortex.2018.01.017 29506747 10.1016/j.cortex.2018.01.017

[26] Audrain, S., & McAndrews, M. P. (2019). Cognitive and functional correlates of accelerated long-term forgetting in temporal lobe epilepsy. *Cortex,**110*, 101–14. 10.1016/j.cortex.2018.03.022 29703447 10.1016/j.cortex.2018.03.022

[27] Rocca, M. A., Barkhof, F., De Luca, J., Frisen, J., Geurts, J. J. G., Hulst, H. E., et al. (2018). The hippocampus in multiple sclerosis. *Lancet Neurology,**17*, 918–26. 10.1016/S1474-4422(18)30309-0 30264730 10.1016/S1474-4422(18)30309-0

[28] Kinsinger, S. W., Lattie, E., & Mohr, D. C. (2010). Relationship between depression, fatigue, subjective cognitive impairment, and objective neuropsychological functioning in patients with multiple sclerosis. *Neuropsychology,**24*, 573–80. 10.1037/a0019222 20804245 10.1037/a0019222 PMC2933087

[29] Carnicka, Z., Kollar, B., Siarnik, P., Krizova, L., Klobucnikova, K., & Turcani, P. (2015). Sleep disorders in patients with multiple sclerosis. *Journal Of Clinical Sleep Medicine*, *11*, 553–557. 10.5664/jcsm.4702 25700869 10.5664/jcsm.4702 PMC4410929

[30] Mayer, G., Happe, S., Evers, S., Hermann, W., Jansen, S., Kallweit, U., et al. (2021). Insomnia in neurological diseases. *Neurol Res Pract*, *3*, 15. 10.1186/s42466-021-00106-3 33691803 10.1186/s42466-021-00106-3 PMC7944611

[31] Wu, W., Francis, H., Lucien, A., Wheeler, T-A., & Gandy, M. (2025). The Prevalence of Cognitive Impairment in Relapsing-Remitting Multiple Sclerosis: A Systematic Review and Meta-analysis. *Neuropsychology Review*, *35*, 233–253. 10.1007/s11065-024-09640-8 38587704 10.1007/s11065-024-09640-8 PMC12328523

[32] Linker, R. A., & Chan, A. (2019). Navigating choice in multiple sclerosis management. *Neurol Res Pract*, *1*, 5. 10.1186/s42466-019-0005-5 33324871 10.1186/s42466-019-0005-5 PMC7650058

[33] Reetz, K., Liepelt-Scarfone, I., Hager, A., Floel, A., Schulz, J. B., Dementia Commission of the German Society for N. (2026). The best treatment is prevention: Prevention of cognitive decline and dementia - current state, gaps and next steps. *Neurology Research and Practice*. 10.1186/s42466-026-00494-4 10.1186/s42466-026-00494-4 PMC13101104 42015253

[34] DeLuca, J., Gaudino, E. A., Diamond, B. J., Christodoulou, C., & Engel, R. A. (1998). Acquisition and storage deficits in multiple sclerosis. *Journal Of Clinical And Experimental Neuropsychology*, *20*, 376–390. 10.1076/jcen.20.3.376.819 9845164 10.1076/jcen.20.3.376.819

[35] Rao, S. M., Leo, G. J., Bernardin, L., & Unverzagt, F. (1991). Cognitive dysfunction in multiple sclerosis. I. Frequency, patterns, and prediction. *Neurology*, *41*, 685–691. 10.1212/wnl.41.5.685 2027484 10.1212/wnl.41.5.685

[36] Artemiadis, A., Bakirtzis, C., Ifantopoulou, P., Zis, P., Bargiotas, P., Grigoriadis, N., et al. (2020). The role of cognitive reserve in multiple sclerosis: A cross-sectional study in 526 patients. *Mult Scler Relat Disord*, *41*, 102047. 10.1016/j.msard.2020.102047 32182469 10.1016/j.msard.2020.102047

[37] Abdulrab, K., & Heun, R. (2008). Subjective memory impairment. A review of its definitions indicates the need for a comprehensive set of standardised and validated criteria. *European Psychiatry,**23*, 321–30. 10.1016/j.eurpsy.2008.02.004 18434102 10.1016/j.eurpsy.2008.02.004

[38] Tullman, M. J. (2013). Overview of the epidemiology, diagnosis, and disease progression associated with multiple sclerosis. *The American Journal Of Managed Care*, *19*, 15–20. 23544716

[39] Strober, L. B., Christodoulou, C., Benedict, R. H., Westervelt, H. J., Melville, P., Scherl, W. F., et al. (2012). Unemployment in multiple sclerosis: The contribution of personality and disease. *Multiple Sclerosis,**18*, 647–53. 10.1177/1352458511426735 22183935 10.1177/1352458511426735

