# Supplementary material for: Accelerated long-term forgetting as an objective marker of subjective memory impairment in multiple sclerosis
Source: Neurol Res Pract. 2026 Aug 24;8(1):69. doi: 10.1186/s42466-026-00527-y (PMC13504967; doi:10.1186/s42466-026-00527-y)
Supplement: Supplementary file 1 [file 42466_2026_527_MOESM1_ESM.pdf]

Supplementary Material

Neurological Research and Practice

Accelerated long-term forgetting as an objective marker of subjective memory impairment in multiple sclerosis

Christina Jansen[1], Johannes Stalter[1,2], Sigrid Reuter[2], Karsten Witt[1,2,3]

[1] Department of Neurology, Faculty of Medicine and Health Science, Carl von Ossietzky University of Oldenburg, Heiligengeisthöfe 4, 26121 Oldenburg, Germany

[2] Department of Neurology, Evangelical Hospital, Steinweg 13, 26122 Oldenburg, Germany

[3] Research Center of Neurosensory Science, Carl von Ossietzky University of Oldenburg, Carl-von-Ossietzky-Straße 9-11, 26129 Oldenburg, Germany

Corresponding Author

Christina Jansen

E-Mail: christina.jansen@uni-oldenburg.de

Table A.1 Disease - modifying medication in multiple sclerosis patients

Medication	MS patients (n, %)
Efficacy category 1 (30-50% reduction in relapse rate)	
Dimethyl fumarate	9 (14.52)
Glatiramer acetate	7 (11.29)
Teriflunomide	3 (4.84)
Interferon-beta 1a	1 (1.61)
Diroximel fumarate	1 (1.61)
Efficacy category 2 (50-60% reduction in relapse rate)	
-	-
Efficacy category 3 (>60% reduction in relapse rate)	
Ocrelizumab	14 (22.58)
Ofatumumab	10 (16.13)
Natalizumab	2 (3.23)

Efficacy categories were adapted from the German guideline recommendation and classified according to relapse rate reduction compared with placebo, modified 2025 [1]. MS multiple sclerosis; n number of patients

Table A.2 Mean scores across RAVLT trials

	MS group (mean, SD)	HC group (mean, SD)	p-value (MWU)
Trial 1	7.68 (1.89)	7.58 (1.80)	0.853
Trial 2	10.66 (2.16)	10.61 (2.16)	0.773
Trial 3	12.27 (1.88)	12.06 (1.98)	0.530
Trial 4	12.79 (1.80)	12.92 (1.84)	0.646
Trial 5	13.61 (1.51)	13.35 (1.72)	0.407
Interference List B	6.35 (1.98)	6.69 (2.06)	0.335
Trial 6 (Post - Interference Recall)	12.11 (2.27)	11.89 (2.57)	0.924

RAVLT Rey's Auditory Verbal Learning Test; MS multiple sclerosis; HC healthy controls; SD standard deviation; MWU Mann-Whitney-U-test

Table A.3 Questionnaires and other test results

	MS group	HC group	p-value
PSQI (SD)	6.47 (3.11)	4.69 (2.25)	0.002 (MWU)
FIS-D (SD)	45.90 (29.50)	22.25 (17.68)	< 0.001 (MWU)
ESS (SD)	6.73 (4.13)	5.32 (2.95)	0.078 (MWU)
SMI (yes/no)	22/40	7/58	0.002 (Chi^2-test)
SMI (0 – 100; SD)	37.26 (16.51)	29.23 (15.34)	0.003 (MWU)
EMQ-S (SD)	31.31 (20.47)	21.63 (15.92)	0.004 (MWU)
FEDA (SD)	92.48 (24.27)	101.40 (20.74)	0.053 (MWU)
FEAG (SD)	90.65 (18.81)	79.83 (14.35)	0.001 (MWU)
MoCA (SD)	28.71 (1,25)	28.77 (1.28)	0.703 (MWU)
SDMT (SD)	55.51 (10.97)	55.31 (11.60)	0.996 (MWU)
BDI-II (SD)	6.13 (4.60)	3.88 (3.79)	0.006 (MWU)

MS multiple sclerosis; HC healthy controls; SD standard deviation; MWU Mann-Whitney-U-test; PSQI Pittsburgh Sleep Quality Index; FIS-D Fatigue Impact; ESS Epworth Sleepiness Scale; SMI Subjective Memory Impairment; EMQ-S Everyday Memory Questionnaire; FEDA Questionnaire of Experienced Deficits of Attention; FEAG Everyday Memory Assessment Questionnaire; MoCA Montreal Cognitive Assessment; SDMT Symbol Digit Modalities Test; BDI-II Beck-Depression-Inventory II

References

1. Hemner B, Gehring K, et al. Diagnose und Therapie der Multiplen Sklerose, Neuromyelitis-optica-Spektrum-Erkrankung (NMOSD) und MOG-IgG-assoziierten Erkrankung (MOGAD), Sk2-Leitlinie. Leitlinien für Diagnostik und Therapie in der Neurologie: Deutsche Gesellschaft für Neurologie; 2026. Available from: https://www.dgn.org/leitlinien (accessed 05 Aug 2026)
